# Coupling of Two Non-processive Myosin 5c Dimers Enables Processive Stepping along Actin Filaments

**DOI:** 10.1038/srep04907

**Published:** 2014-05-09

**Authors:** Laura K. Gunther, Ken'ya Furuta, Jianjun Bao, Monica K. Urbanowski, Hiroaki Kojima, Howard D. White, Takeshi Sakamoto

**Affiliations:** 1 Department of Physics and Astronomy, Wayne State University, Detroit, MI 48201, USA; 2 Department of Physiology, Wayne State University School of Medicine, Detroit, MI 48201, USA; 3 Advanced ICT Research Institute, National Institute of Information and Communications Technology, Kobe 651-2492, Japan; 4 Department of Physiological Sciences, Eastern Virginia Medical School, Norfolk, VA 23507, USA

## Abstract

Myosin 5c (Myo5c) is a low duty ratio, non-processive motor unable to move continuously along actin filaments though it is believed to participate in secretory vesicle trafficking in vertebrate cells. Here, we measured the ATPase kinetics of Myo5c dimers and tested the possibility that the coupling of two Myo5c molecules enables processive movement. Steady-state ATPase activity and ADP dissociation kinetics demonstrated that a dimer of Myo5c-HMM (double-headed heavy meromyosin 5c) has a 6-fold lower Km for actin filaments than Myo5c-S1 (single-headed myosin 5c subfragment-1), indicating that the two heads of Myo5c-HMM increase F-actin-binding affinity. Nanometer-precision tracking analyses showed that two Myo5c-HMM dimers linked with each other via a DNA scaffold and moved processively along actin filaments. Moreover, the distance between the Myo5c molecules on the DNA scaffold is an important factor for the processive movement. Individual Myo5c molecules in two-dimer complexes move stochastically in 30–36 nm steps. These results demonstrate that two dimers of Myo5c molecules on a DNA scaffold increased the probability of rebinding to F-actin and enabled processive steps along actin filaments, which could be used for collective cargo transport in cells.

Cargo transport is an essential cellular process important for many physiological functions of cells. Class 5 myosins are actin-based molecular motors implicated in organelle transport^1^,^2^,^3^,^4^. Like other members of the myosin superfamily, myosin 5 consists of three major domains^1^. The motor domain at the amino terminus contains the binding sites for nucleotide and actin. It is followed by an elongated neck domain consisting of a single α-helix of the heavy chain that contains six IQ motifs, each of which binds to a calmodulin or a calmodulin family member^5^. Following the neck region, the heavy chain contains significant stretches of predicted coiled-coil sequence and a carboxyl terminal globular tail domain. Two heavy chains are dimerized by the coiled-coil region to form a two-headed molecule.

The importance of myosin 5 in intracellular transport has been well studied^1^,^2^,^3^,^4^,^6^. In yeast, two classes of myosin 5, Myo2p and Myo4p, have been identified^7^,^8^,^9^,^10^. Myo2p is directly involved in polarized transport of secretory vesicles^11^ and peroxisomes^12^. In addition, it is involved in the transportation of Golgi apparatus and vacuoles^7^,^13^ and in the orientation of the mitotic spindle^7^,^10^. Myo4p is known to transport at least two mRNAs, ASH1 and IST2, to the bud tip^9^,^14^,^15^. In vertebrates, there are three myosin 5 genes encoding three isoforms of myosin 5, referred to as myosin 5a, 5b, and 5c. Myosin 5a is abundantly expressed in the skin^16^,^17^,^18^ and brain^19^,^20^, and is the best characterized isoform for cargo transport^17^,^21^,^22^. In the melanocytes of the skin, myosin 5a was found to transport melanosomes via interaction with RAB27 and melanophilin^23^,^24^. In the brain, recent studies demonstrated that myosin 5a transports the endoplasmic reticulum (ER) within the dendritic spines of Purkinje neurons^22^. Myosin 5b is mainly expressed in non-neuronal tissues and has been shown to transport recycling endosomes and cytoplasmic vesicles^25^,^26^. By contrast, Myo5c is abundant in epithelial and glandular tissues^3^,^27^,^28^ and was found to be associated with secretary vesicles^3^. Recent studies have shown that myosin 5c participates in apical exocytosis in lacrimal acinar cells^27^,^29^,^30^ though the exact function of myosin 5c during this process is unknown. To date, there is no clear evidence that myosin 5c functions as an intracellular transporter.

For a single dimer of myosin 5 to be an effective cargo transporter, it is important that the reattachment rate of its leading head to actin (Actin + M-ADP-Pi → ActoM-ADP-Pi → AM-ADP) is much faster than the dissociation rate of the rear head. In this manner, at least one of the two heads remains attached, allowing continuous movement of the motor molecule along the actin filament. This type of behavior is termed “processive movement”. The kinetic properties of myosin 5a make it an ideal molecule to achieve such processive movements. Kinetics studies using single-headed Myo5a-S1 fragment reveal that it is a high duty ratio motor, meaning that it spends most of its ATPase cycle in a strong-binding state with F-actin. The rate-limiting step during the ATPase cycle is ADP dissociation from the motor head, leading to the detachment of the motor head from the actin filaments^31^. Further study using dimeric myosin 5a heavy meromyosin (HMM) fragment showed biphasic kinetics of ADP release in the presence of actin when both heads were strained by simultaneous binding to an single actin filament^32^. This demonstrates that strain resulting from the two heads of myosin 5a binding to actin increases the run length by reducing the rate of ADP dissociation from the leading head. This head-to-head gating (or gaited-gating) mechanism could reduce the probability of simultaneous detachment of both head from actin filament and therefore increase the run length. Single-molecule experiments including optical trapping^33^,^34^,^35^,^36^, total internal reflection fluorescence (TIRF) microscopy^37^,^38^,^39^,^40^,^41^, and atomic force microscopy (AFM)^42^ have demonstrated that myosin 5a takes 36 nm steps in a hand-over-hand fashion along actin filaments during processive movement. In contrast, unlike myosin 5a, transient solution kinetics studies showed that single-headed myosin 5c S1 (Myo5c-S1) has a low duty ratio (~20%), spending most of its ATPase cycle (~80%) in a weak actin-binding state^43^,^44^, increasing the probability that both heads detach at the same time. Consistent with its biochemical properties, single molecule studies confirmed that Myo5c is a non-processive motor, i.e. a single Myo5c molecule does not continuously move along actin filaments^43^,^44^.

In this study, we performed the kinetic study on double-headed Myo5c-HMM fragment and examined the processivity of two Myo5c-HMM molecules that are linked together on a DNA scaffold. We show that, similar to myosin 5a, the ADP dissociation of Myo5c-HMM is biphasic, and the duty ratio (40%) of Myo5c-HMM is higher than that of Myo5c-S1, but these properties do not produce processive movement by single molecules. In contrast, we also show that two molecules of Myo5c-HMM dimers linked with each other on a DNA scaffold can move processively along actin filaments.

## Results

### Expression and purification of myosin 5c fragments

**Figure 1:**
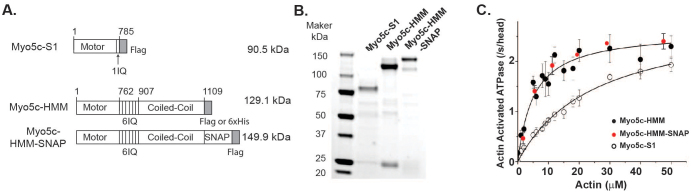
Generation of myosin 5c fragments. (A) Stick diagrams of myosin 5c S1 (Myo5c-S1) and HMM (Myo5c-HMM) fragments without (middle) and with a SNAP-tag (bottom). A FLAG tag or 6xHis tag was fused at the C-terminus of all fragments. The numbers are the positions of human myosin 5c amino acids. The estimated molecular weights are indicated. (B) SDS-PAGE of the purified myosin 5c fragments. (C) The steady-state actin-activated ATPase activity of Myo5c-HMM without (black circles) or with (red circles) SNAP-tag, and Myo5c-S1 (open circles).

The Myo5c-S1 and -HMM fragments with or without a SNAP-tag were generated using the baculovirus Sf9 system (Figure 1A). A FLAG-tag was incorporated into the C-terminus of the proteins to facilitate affinity purification. Myo5c-S1 is a 90.5-kDa single-headed monomer containing only the motor domain and one IQ motif, whereas Myo5c-HMM is a double-headed molecule containing the motor domain, six IQ motifs and a coiled-coil region for dimerization. The estimated molecular weights of the heavy chains of Myo5c-HMM and Myo5c-HMM-SNAP are 130 kDa and 150 kDa, respectively. The purified proteins were confirmed by SDS-PAGE (Figure 1B).

### ATPase activity and actin gliding of Myo5c-HMM and -S1

We first measured the actin-activated ATPase activities of Myo5c-HMM and Myo5c-S1. The maximum ATPase rates (V_max_) of Myo5c-HMM and -S1 were similar (2.31 s^−1^ and 2.76 s^−1^, respectively; Figure 1C and Table 1). However, the K_ATPase_ of Myo5c-HMM was 6-fold smaller than that of Myo5c-S1 (5.1 ± 1.8 vs. 35 ± 3.7 μM, shown as means ± S.D.; Figure 1C and Table 1), indicating that the double-headed Myo5c-HMM exhibits stronger affinity for actin filaments than does the single-headed Myo5c-S1. The V_max_ and K_ATPase_ for Myo5c-HMM-SNAP are similar to the values of Myo5c-HMM (Figure 1C and Table 1), indicating that SNAP-tag does not interfere with the ATPase activity of Myo5-HMM.

Next, we performed actin-gliding assays to assess the motility of multiple Myo5c-HMM molecules at saturating ATP concentration (1 mM). Actin filaments were shown to move effectively over both Myo5c-HMM and -HMM-SNAP with velocities of 73.7 ± 15 and 82.6 ± 26 nm/s, respectively (Supplemental Figure S2B online). The time-lapse images of actin gliding over Myo5c-HMM were shown in Supplemental Figure S2A online.

### ADP dissociation kinetics of actomyosin 5c-HMM and -S1

**Figure 2:**
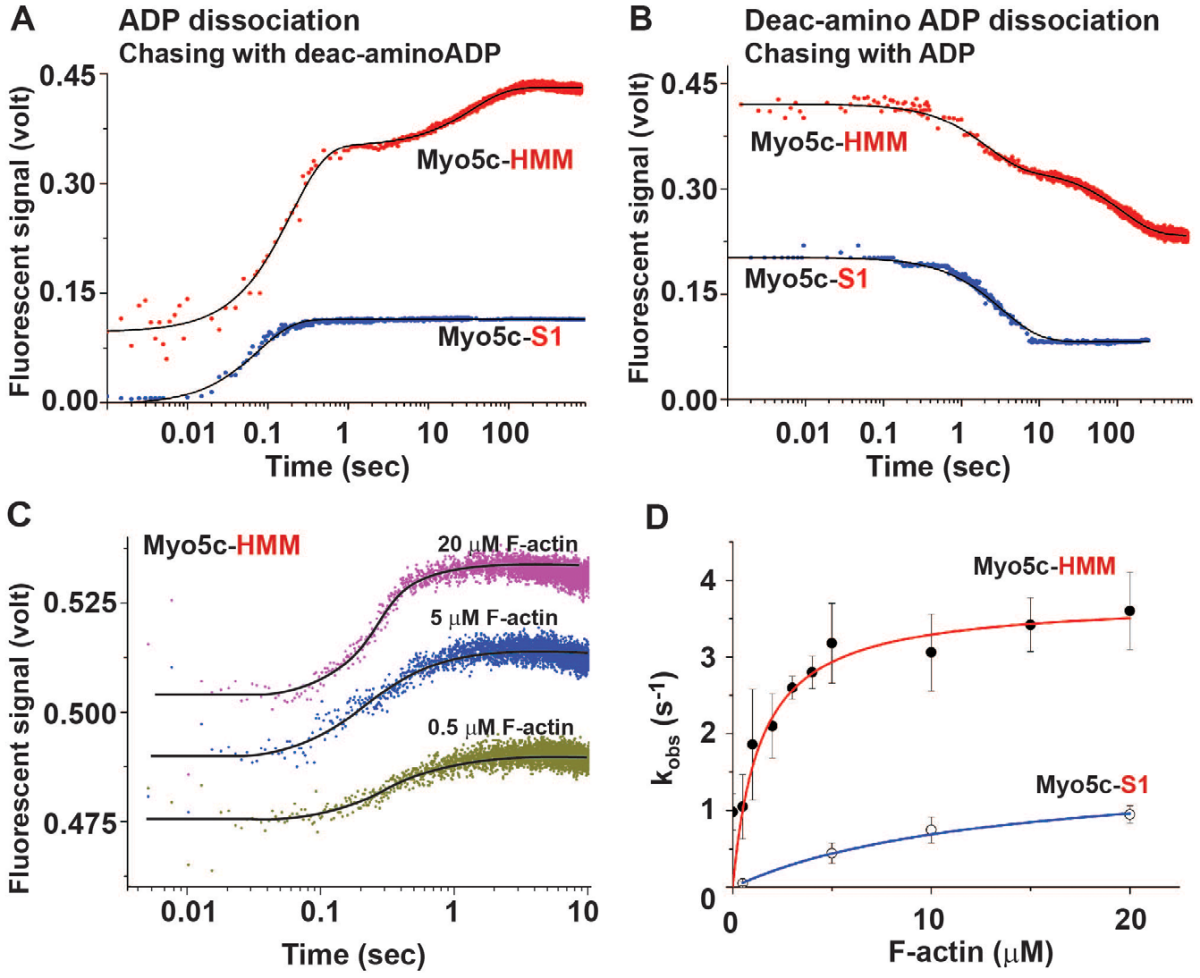
ADP and phosphate dissociation rates of actomyosin 5c-HMM and -S1. (A and B) The typical traces of ADP dissociation (A) and deac-aminoADP dissociation (B) from Myo5c-HMM and S1 were done as described in Methods. (C) Phosphate dissociation events from actomyosin 5c-HMM and -S1 were observed with double-mixing stopped flow measurements. Representative traces for Myo5c-HMM are shown in three different concentrations of F-actin (0.5, 5, and 20 μM). The data were fitted with a single exponential function. (D) Phosphate dissociation rates (k_obs_, s^−1^) of Myo5c-HMM (closed circle) and -S1 (open circle) are plotted against varying F-actin concentrations. The rates were averaged with three different experiments conducted on different days. The data were fitted with a hyperbolic function and were averaged from three independent experiments. The saturated phosphate release rates of Myo5c-HMM and -S1 were 3.67 ± 0.7 (s^−1^) and 1.7 ± 0.4 (s^−1^), respectively. The half-maximum values of Myo5c-HMM and S1 were 1.1 ± 0.2 μM and 14.6 ± 1.2 μM, respectively.

Although previous kinetics studies revealed a low duty ratio (16–33%) of the single-headed Myo5c-S1^43^,^44^, our ATPase assay demonstrated a higher F-actin affinity of double-headed Myo5c-HMM than that of single-head Myo5c-S1 (Figure 1C), raising the possibility that the ATPase kinetics of double-headed Myo5c-HMM may be different from that of Myo5c-S1. As such, we used double-mixing stopped-flow methods to compare the rate constants of ADP dissociation from actomyosin 5c-HMM-ADP-Pi or 5c-S1-ADP-Pi complex. The large increase in fluorescence signals observed when deac-aminoADP binds to the motor domain of Myo5c-HMM and -S1 was used to measure ADP dissociation (Figure 2A). The data for Myo5c-HMM were fitted with the double exponential function with fast and slow phases having amplitudes of 0.19 and of 0.12 for Myo5c-HMM, respectively (Eq. 2, see Materials and methods). Similar biphasic ADP dissociation rates were obtained with Myo5c-HMM as previously observed with Myo5a-HMM. ADP dissociation from the trailing head of Myo5c-HMM was faster (5.5 ± 0.2 s^−1^) than from the leading head (0.026 ± 0.009 s^−1^) with amplitudes of 0.13 and 0.09, respectively (Figure 2A; red circle and Table 2). In contrast, the data for Myo5c-S1 were fitted with a single exponential function, and the rate was 14.3 ± 0.9 s^−1^ with amplitude of 0.09 (Figure 2A; blue circle and Table 2). The amplitudes of the fast phase of both ADP and deac-aminoADP dissociation from Myo5c-HMM are 10–20% larger than those of the slow phase. Myo5c-HMM-SNAP has the same rate of ADP and deac-aminoADP dissociation as Myo5c-HMM does (Table 2). However, when excess deac-aminoATP was used instead of deac-aminoADP in syringe 3 (Supplemental Figure S2A online), the data were fitted with single exponential function of 3.9 ± 0.4 s^−1^, similar to the fast rate of ADP dissociation of actomyosin 5c-HMM with a deac-aminoADP chase.

We also did experiments to measure deac-aminoADP dissociation from actomyosin 5c-HMM-deac-aminoADP-Pi and 5c-S1-deac-aminoADP-Pi complexes in which Myo5c-HMM and -S1 were mixed with deac-aminoATP followed by addition of excess ADP (1 mM) and phalloidin-labeled F-actin (Figure 2B). In these experiments, the fluorescence signals were decreased as deac-aminoADP dissociated from the ATP binding site of the myosin motor domain. The data for Myo5c-HMM and -S1 were fitted with double and single exponential equations (3) and (2), respectively. Similar to ADP dissociation, deac-aminoADP dissociation from actomyosin Myo5c-HMM-deac-aminoADP-Pi was also biphasic (Figure 2B; red circle and Table 2) with fast (0.31 ± 0.09 s^−1^) and slow rates (0.0086 ± 0.0021 s^−1^). However, when ATP instead of ADP was used to chase deac-aminoADP from Myo5c-HMM-deacaminoADP-Pi (Supplemental Figure S2B online), the data were fitted by a single exponential function with a rate constant of 0.29 ± 0.09 s^−1^. The dissociation of the trailing head by ATP releases the strain on the leading head allowing the deac-aminoADP to dissociate from the leading head at the unstrained rate. In contrast, the deac-aminoADP dissociation rate of Myo5c-S1 showed a single phase of 1.25 ± 0.21 s^−1^ (Figure 2B, blue circles). It is of note that the rates of ADP and deac-aminoADP dissociation from Myo5c-S1 were 3–4 times faster than the fast rates of Myo5c-HMM. This suggests that the strain between the two heads of Myo5c-HMM reduces the rates of ADP and deac-aminoADP dissociation from the trailing head of actoMyo5c-HMM-ADP-Pi, but by a smaller amount than from the leading head. In addition, the ADP dissociation rates were about 15 times faster than the deac-aminoADP dissociation rates, which was also observed in myosin 5a^32^. Taken together, the above results demonstrate that double-headed Myo5c-HMM has slower ADP dissociation rate than single-headed Myo5c-S1, which is expected to increase the duty ratio of Myo5c dimers (see below).

### Phosphate releasing kinetics of Myo5c-HMM and -S1

The rate of phosphate (Pi) dissociation from actomyosin5c-HMM-ADP-Pi or -S1-ADP-Pi was monitored by using MDCC-PBP (Figure 2C)^32^,^45^. The maximum value of k_obs_ and the half-maximum value (K_act_) for Myo5c-HMM were 3.7 ± 0.7 s^−1^ and 1.1 ± 0.2 μM, respectively, while the rates for Myo5c-S1 were 1.7 ± 0.4 s^−1^ and 14.6 ± 1.2 μM (Figure 2D). These rates measured with Myo5c-S1 were consistent with previous studies^43^,^44^.

### Duty ratio of Myo5c-HMM and -S1

**Figure 3:**
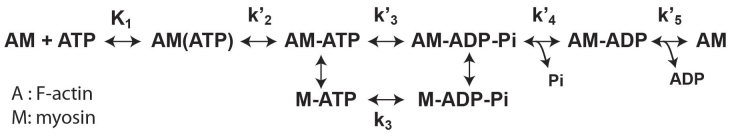
The myosin/actomyosin ATPase cycle. A: actin, M: myosin.

The duty ratio of ATPase kinetics can be calculated from the phosphate and ADP dissociation rates, which represent the rates of transition between weak- and strong-binding states during the actomyosin ATPase cycle, respectively. The duty ratio was calculated by: 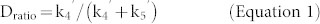
where k_4_' is the phosphate dissociation rate, and k_5_' is the ADP dissociation rate (Figure 3). k_4_' for Myo5c-HMM and -S1 were 3.7 ± 0.7 s^−1^ and 1.7 ± 0.5 s^−1^, respectively (Figure 2C). The k_5_' for Myo5c-HMM and -S1 were 5.5 ± 0.2 s^−1^ and 14.3 ± 0.3 s^−1^, respectively (Figure 2A). Thus, the duty ratios of Myo5c-HMM and Myo5c-S1 were calculated to be 40.2% and 10.6%, respectively. The duty ratio of Myo5c-HMM was much higher than that of Myo5c-S1, suggesting that the double-heads of Myo5c coordinate with each other to decrease the rate of ADP dissociation and increase the rate of phosphate dissociation, which increases the duty ratio.

### The stepping of a single Myo5c-HMM molecule along actin filaments

**Figure 4:**
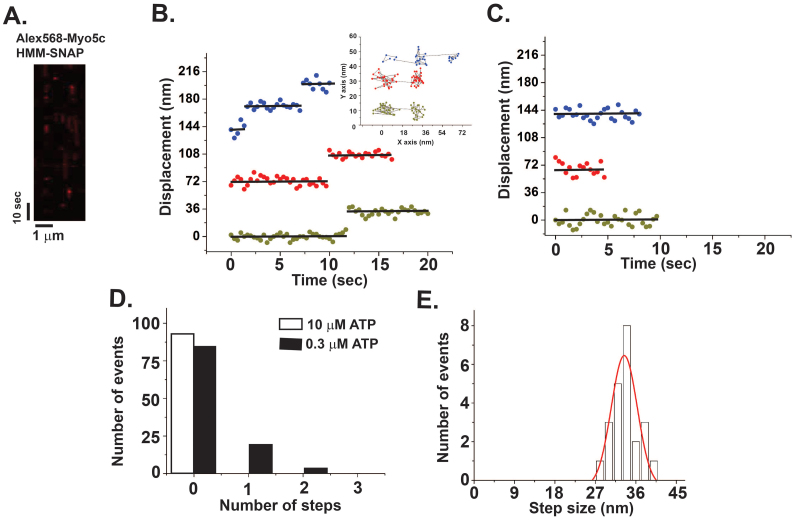
The movement of a single Myo5c-HMM-SNAP dimer. (A) Kymograph of single-molecule motility assay for Alexa Fluor 568-labeled Myo5c-HMM-SNAP. Myo5c molecule (red spot) binds to and dissociates from F-actin. (B and C) Typical stepping traces of Myo5c-HMM-SNAP in the presence of 10 μM or 0.3 μM ATP plotted as a function of time. Average positions were represented as black lines. Inset shows the XY-position of the three spots. Each color in B represents the same trace. (D) Number of steps of Myo5c-HMM-SNAP in the presence of 0.3 μM ATP (n = 103, closed bar) and 1 μM ATP (n = 92, open bar). (E) Step-size histogram of Myo5c-HMM-SNAP. The data (n = 21) were fitted with Gaussian function (red line). The average step-size was 34.5 ± 5.5 nm (mean ± S.D.).

To test whether a single Myo5c-HMM molecule moves along actin filaments, we first performed motility assays of Alexa Fluor 568-labeled single Myo5c-HMM dimer by TIRF microscopy. At higher ATP concentration (>10 μM), we did not observe any processive movements of Myo5c-HMM (Figure 4A and Movie 1). However, at 300 nM ATP, 18% of Myo5c-HMM molecules (total number of observed spots: 103) showed one or two steps with an average step size of 34.5 ± 5.5 nm (Figure 4B), while 82% of the spots did not show any visible stepping events (Figure 4C). The non-stepping molecules only showed binding events without processive stepping along actin filaments (Figure 4A, C and D; open bar). 16% of Myo5c-HMM-SNAP molecules also took one or two steps. The XY plot in the inset of Figure 4B shows that the observed steps are forward stepping rather than backward stepping. These results demonstrate that Myo5c is a non-processive motor in a physiological ATP concentration (1 mM ATP), which is consistent with our kinetics results and previous single-molecule studies^43^,^44^. However, a single Myo5c-HMM molecule can take a few steps at lower ATP concentration when the ATP binding is a rate-limiting step.

### Coupling of two Myo5c dimers on a DNA scaffold

**Figure 5:**
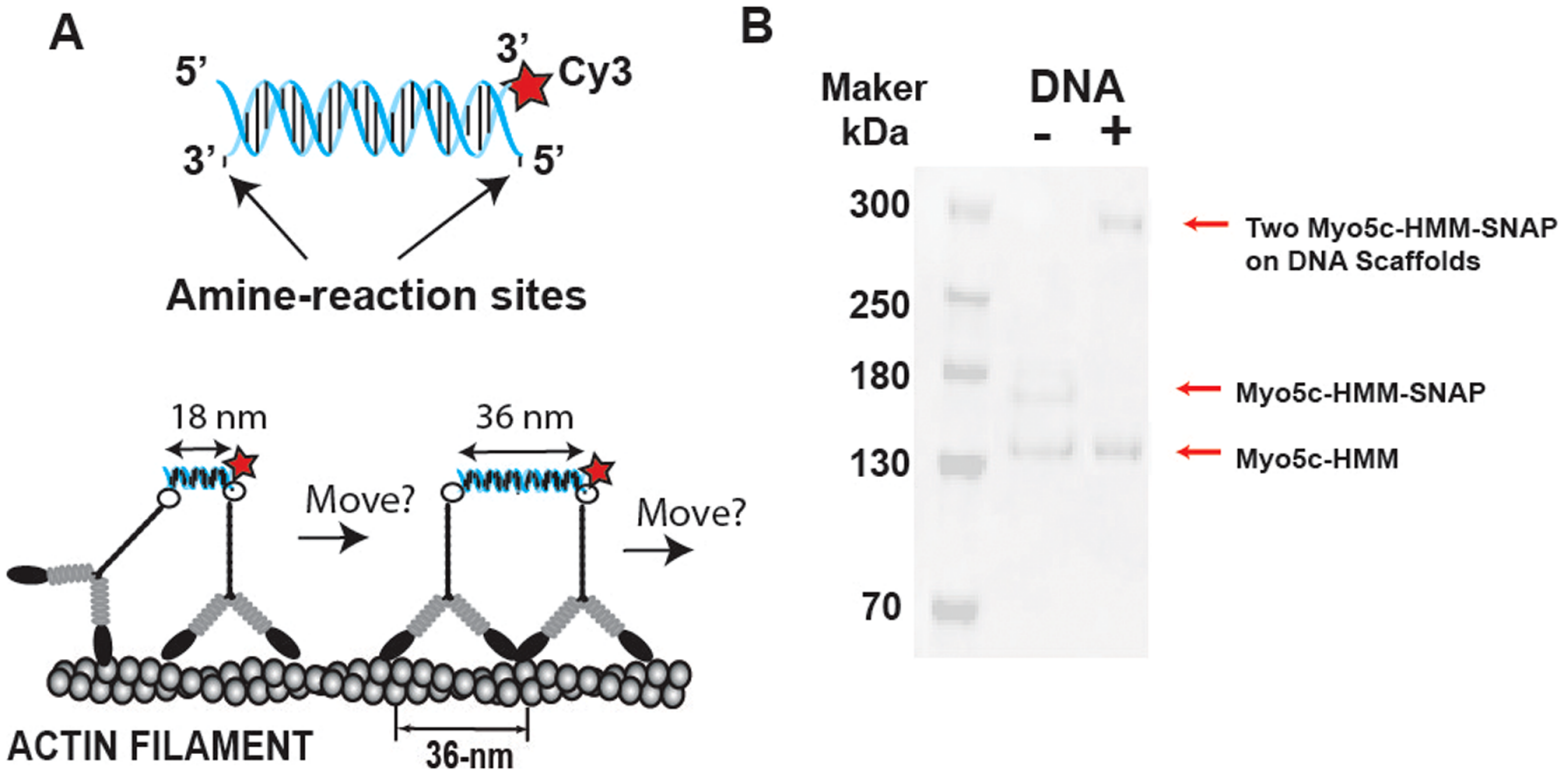
Schematic of DNA scaffolds conjugated with myosin 5c-HMM molecules. (A) Top: schematic of the DNA scaffold used in this study. One DNA strand has amine-reactive sites at both 3′ and 5′ ends (arrow), and the other strand is labeled with Cy3 at 5′ end (red star). Bottom: the schematic of two myo5c-HMM molecules conjugated to a DNA scaffold with two different motor-motor distances (18 and 36 nm). (B) SDS-PAGE of Myo5c-HMM in the absence (-) or presence (+) of a DNA scaffold. Arrows indicate the bands of one Myo5c-HMM, Myo5c-HMM-SNAP, and two Myo5c-HMM molecules on a DNA scaffold.

To test the possibility that multiple Myo5c dimers move processively, we engineered a Myo5c-HMM complex containing two Myo5c-HMM dimers using DNA scaffolds (Figure 5A), a recently developed technique that has been used to engineer nanostructures with pre-designed geometries^46^,^47^. We used double-stranded DNA scaffolds, of which one strand was amine-modified at both ends for interacting with two Myo5c-HMM molecules, and the other strand was labeled with Cy3 dye at 5′ end for fluorescence imaging (Figure 5A, top panel). The resultant myosin-DNA complexes with three different motor-motor distance on a DNA scaffold (6.8, 18, and 36nm) are hereafter referred to as Myo5c-HMM-dDNA_6.8_, Myo5c-HMM-dDNA_18_ and Myo5c-HMM-dDNA_36_, respectively (Figure 5A, bottom panel). One heavy chain of the heterodimer Myo5c-HMM has a SNAP-tag at the C-terminus, whereas the other does not. The success of conjugating two hetero-Myo5c-HMM dimers (i.e. Myo5c-HMM-SNAP and Myo5c-HMM) onto a DNA scaffold, which is referred to as Myo5c-HMM-dDNA, was verified by SDS-PAGE (Figure 5B). There is no obvious difference in the molecular weights of Myo5c-HMM-dDNA_6.8_, Myo5c-HMM-dDNA_18_, and Myo5c-HMM-dsDNA_36 _on SDS-PAGE gels.

### The processivity and step size of the two coupled myosin 5c molecules

To test the activity of myosin 5c conjugated with DNA scaffolds, Myo5c-HMM-dDNA-Cy3 complexes were bound on Alexa Fluor 488 phalloidin-labeled actin filaments. The fluorescence signals of Alexa Fluor 568 or Cy3-labeled myosin overlapping with Alexa Fluor 488-phalloidin-labeled actin filaments were observed under a TIRF microscope. After 1 mM ATP in motility buffer was added into the motility chamber, the extent of Myo5c dissociated from actin filaments was assessed. If the myosin molecules are active, a decreased overlap signal of Alexa Fluor 568 or Cy3 with Alexa Fluor 488 will be observed as a result of the detachment of myosin from actin filaments by ATP. On the contrary, if Myo5c-HMM molecules are denatured or dead, the fluorescence signals from myosin on actin filaments could still be observed. We used only Myo5c-HMM-dDNA preparations in which most of the Cy3 signal dissociated from actin filaments upon addition of ATP, i.e. their ATPase activity was well preserved.

**Figure 6:**
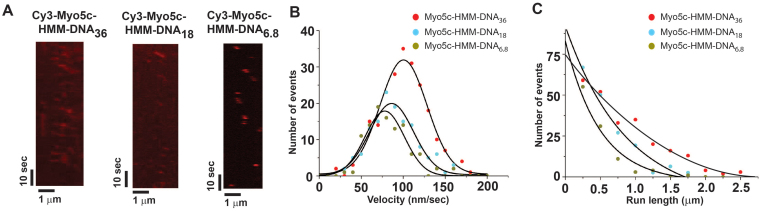
Velocity and run length of two Myo5c dimers coupled on a DNA scaffold. (A) Kymographs depicting the movements of Cy3-Myo5c-HMM-DNA_36_, Cy3-Myo5c-HMM-dDNA_18_, and Cy3-Myo5c-HMM-dDNA_6.5_. Red spots represent Myo5c-DNA complexes. (B) Histogram of the velocity of Myo5c-HMM-dDNA_36_ (red circles), Myo5c-HMM-dDNA_18_ (cyan circles), and Myo5c-HMM-dDNA_6.8_ (dark yellow circles). The data were fitted with a single Gaussian function. The peak of the velocity of Myo5c-HMM-dDNA_36_, Myo5c-HMM-dDNA_18_, and Myo5c-HMM-dDNA_6.8_ was 100.3 ± 32.4 nm/sec (n = 232), 86.2 ± 31 nm/s (n = 135), and 78.5 ± 23.3 nm/sec (n = 105), respectively. (C) Histograms of the run length of Myo5c-HMM-dDNA_36_ (red circles), Myo5c-HMM-dDNA_18_ (cyan circles), and Myo5c-HMM-dDNA_6.8_ (dark yellow circles). The data were fitted with a single exponential function. The average run length of Myo5c-HMM-dDNA_36_ (red circles), Myo5c-HMM-dDNA_18_ (cyan circles), and Myo5c-HMM-dDNA_6.8_ (dark yellow circles) was 1755 ± 210 nm (n = 232), 680 ± 55 nm (n = 135), and 497 ± 92 nm (n = 105), respectively.

We next tested whether the two Myo5c-HMM dimers coupled on a DNA scaffold were able to move processively along actin filaments at physiological ATP concentration (1 mM) by using TIRF microscopy. Unlike single Myo5c-HMM molecules, which do not exhibit detectable processive movement along actin filaments, all complexes, Myo5c-HMM-dDNA_36_, Myo5c-HMM-dDNA_18_, and Myo5c-HMM-dDNA_6.8_, move processively along actin filaments at 1 mM ATP. The velocities of Myo5c-HMM-dDNA molecules decrease slightly with shorter lengths of DNA scaffold with an average velocity of 100.3 ± 32.4 nm/s (movie 2), 86.2 ± 31 nm/s (movie 3), and 78.5 ± 23 nm/s (movie 4), respectively (Figure 6A and B). The run length was also decreased with shorter length of DNA scaffold (Myo5c-HMM-dDNA_36_: 1755 ± 210 nm, Myo5c-HMM-dDNA_18_: 680 ± 55 nm, and Myo5c-HMM-dDNA_6.8_: 497 ± 92 nm, Figure 6C), indicating that the distance between the two Myo5c-HMM dimers affects their run length (see discussion).

**Figure 7:**
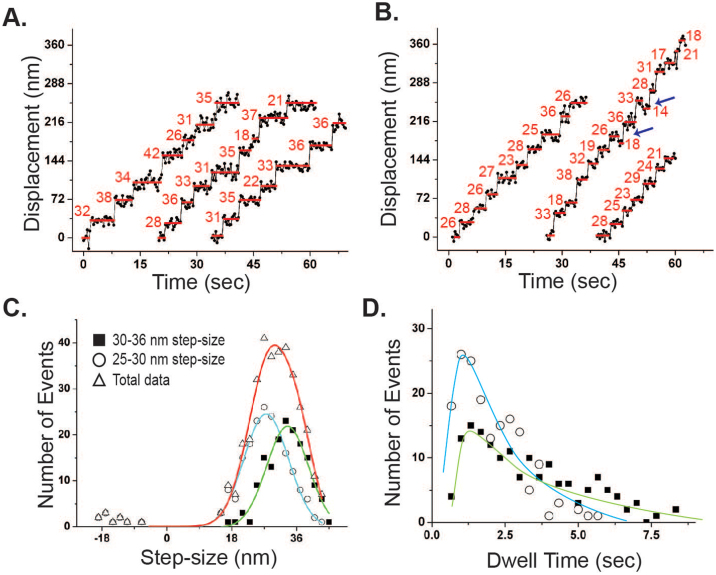
The stepping traces and the step-size histograms of two Myo5c-HMM dimers conjugated on dDNA_36_ scaffolds. (A and B) Stepping traces of 30-36 nm and 25–30 nm step-sizes of Myo5c-HMM-dDNA_36_. The numbers (red) are calculated step-sizes. Blue arrows represent backwasd steps of Myo5c-DNA complex. (C) The step-size histograms of overall stepping (open triangle and red solid line), 25–30 nm stepping (open circle and light blue solid line), and 30-36 nm stepping (closed square and green solid line). Data were fitted with a single Gaussian function. The average step-size of overall, 25–30 nm, and 30–36 nm stepping of Myo5c-HMM-DNA_36_ was 30.4 ± 7.9 nm (n = 342), 27.5 ± 6.7 nm (n = 187), and 33.5 ± 6.4 nm (n = 155), respectively. Data represent means ± S.D. (D) Dwell time histograms of 30–36 nm (closed square and green solid line) and 25–30 nm step-size (open circle and light blue solid line) were fitted with P(t) = tk^2^e^−kt^. The rate of dwell time of 30–36 nm steps and 25–30 nm steps was 0.63 ± 0.15 s^−1^ and 1.43 ± 0.23 s^−1^, respectively.

The stepping direction and the step-size of the processive movement were measured at 10 μM ATP, which slows down the myosin movement such that the stepping behavior can be analyzed by FIONA. The stepping traces of Myo5c-HMM-dDNA_36_ are shown in Figure 7A. The histogram of the overall step-size of Myo5c-HMM-dDNA_36_ shows a broad distribution with an average of 30.4 ± 7.9 nm (Figure 7C, open triangle with red solid line, mean ± S.D., n = 342). Further analysis of the stepping traces revealed two types of stepping of Myo5c-HMM-dDNA_36_: one with a 30–36 nm step-size and the other with a 25–30 nm step-size, both of which exhibited single Gaussian distributions (Figure 7C, closed square/green line and open circle/blue line, respectively). The average step-sizes for the stepping with 30–36 nm and 25–30 nm were 33.5 ± 6.4 nm (n = 155) and 27.5 ± 6.7 nm (n = 187), respectively. Additionally, the stepping with 25–30 nm step-size also showed backward steps (average −15 ± 3.2 nm), which were about 9% of the total steps (Figure 7B, blue arrows) and were not observed in the 30–36 nm steps.

The dwell time for the 30–36 and 25–30 nm step-sizes were calculated from the stepping traces and were plotted in Figure 7D. Note that, the traces with only one frame were eliminated because of the difficulty in discriminating between a full step and the middle of a step. Hence, the dwell time was calculated from the ones longer than 0.66 sec (i.e. longer than one frame). To analyze the stepping rates from the dwell time histogram, each Myo5c-HMM-dDNA molecule is considered to take a stepping rate, k, so that the stepping rate of two Myo5c-HMM-dDNA molecules can be fitted with identical rate constant. Thus, the derived kinetic equation in this case is P(t) = tk^2^e^−kt^, and was used to fit the data. The stepping rates measured for larger and shorter steps were 0.63 ± 0.15 s^−1^ and 1.43 ± 0.23 s^−1^, respectively.

**Figure 8:**
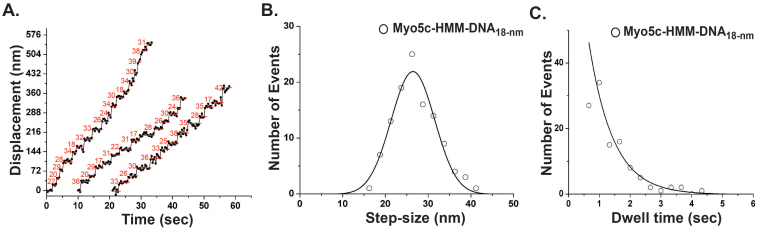
Stepping traces, step-size, and dwell time of two Myo5c-HMM dimers conjugated on a dDNA_18_ scaffold. (A) Stepping traces of Myo5c-HMM-dDNA_18_. The numbers (red) are calculated step-sizes. (B) Step-size histogram. The data of the step-size were fitted with a single Gaussian function. The average step-size was 26.2 ± 7.6 nm (n = 113). (C) The dwell time histogram. The data were fitted with a single exponential function, P(t) = ke^−kt^, where k is a time constant (0.8 s^−1^).

On the other hand, the stepping traces and the histogram of the step size of Myo5c-HMM-dDNA_18_ are shown in Figure 8A and B, respectively. The distribution of the step-size histogram exhibited single Gaussian distribution with an average step-size of 26.2 ± 7.6 nm (n = 113). The stepping traces did not show any obvious separate groups or back steps. The dwell time was well fitted by a single exponential, P(t) = ke^−kt^ with a rate of 0.8 s^−1^ (Figure 8C), which was twice as slow as that of single Myo5c-HMM on actin filaments (1.96 s^−1^)^44^. The stepping mechanism of Myo5c-HMM-dDNA_6.8_ cannot be resolved, because it was difficult to distinguish between the steps of Myo5c-HMM-dDNA_6.8_ (Supplemental Figure S3).

## Discussion

In the current study, we found that the duty ratio of Myo5c-HMM was increased to 40%. However, Myo5c-HMM is still unable to move processively along actin filaments at physiological ATP concentrations. On the other hand, at low ATP concentration (0.3 μM), Myo5c-HMM took one or two steps before dissociating from actin. At such a low ATP concentration, the dissociation rate of Myo5c motor domain from actin is slower than the rate of reattachment, thereby enabling short processive movement. Thus, we hypothesize that multiple Myo5c-HMM molecules bound to a DNA scaffold would prevent the dissociation of Myo5c from actin, which may enable it to take multiple steps along actin filaments.

The molecular complex containing two Myo5c-HMMs with a distance of 36 nm on a DNA scaffold displayed a run length comparable to that of a single myosin 5a molecule. The run length and step-size of two coupled Myo5c-HMM molecules decreased with decreasing spacing between two motors. The velocity is also slightly decreased by a shorter length of DNA. This adaptive feature may be important for Myo5c to adjust its movement and its kinetics to *in vivo* conditions such as the size, shape, and structure of the cargo, as well as its distribution density on the surface of the cargo. Recent studies showed that Myo5c is expressed in epithelial and glandular tissues where it is bound to large-size cargos such as zymogen granules^28^,^30^ (~800 nm in diameter) instead of small cargos as in neurons (~40 nm in diameter)^48^. Thus, we hypothesize that this non-processive feature and adaptable kinetics properties of Myo5c are necessary to transport large-size cargoes along actin filaments, where highly-ordered structures are formed, such as mesh-network and uni-/bi-polar F-actin bundles.

**Figure 9:**
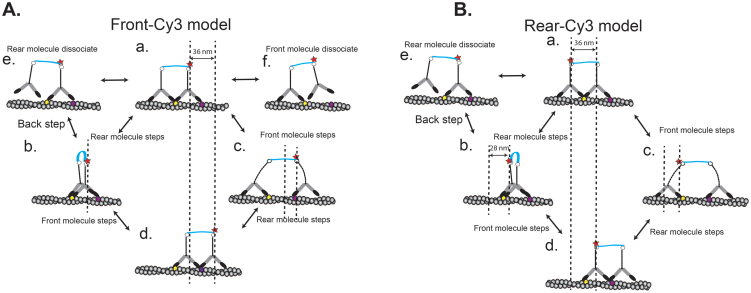
Stepping models of two Myo5c dimers on dDNA_36_ scaffold. (A) The front-Cy3 model in which Cy3 is positioned at the front of the Myo5c-DNA complex. Two stepping pathways (a → b → d and a → c → d) are shown. The pathway of a → b → d shows that when a rear Myo5c molecule steps (a → b), which is a silent step for the complex, then the front Myo5c molecule steps (b → d). The pathway of a → c → d shows that after the front Myo5c molecule steps (a → c), the rear Myo5c molecule steps (c → d). Step (a → c) takes 30–36 nm, while step (c → d) takes a shorter step size (<10 nm). A rear (e) and a front (f) Myo5c molecule dissociate from actin. (B) The rear Cy3 model. This model shows the contribution of the stepping events by both front and rear Myo5c molecules in the complex. The pathway of a → b → d shows that after the rear Myo5c molecule takes a step (a → b), the front Myo5c molecule steps (b → d). The pathway a → c → d represents that the front Myo5c molecule steps first and then the rear Myo5c molecule steps.

Our kinetic studies show that Myo5c-HMM molecule has two ADP dissociation rates, suggesting that Myo5c-HMM takes a “gated-gait” mechanism during steps, e.g. an internal strain in the molecule is created by a conformational change in the front head. As a result, ADP dissociates more rapidly from the trailing head than from the leading head of myosin, allowing the molecule to step forward. Similarly, two Myo5c-HMM molecules attached to a DNA linker are likely to create an internal strain, that is, depending on the stiffness of the DNA linker. To explain these results, we propose a stepping model for Myo5c-HMM-dDNA (Figure 9). Our data with 36-nm length of DNA linker (Myo5c-HMM-dDNA_36_) revealed two populations with different step-sizes (25–30 nm and 30–36 nm). We reasoned that during the processive movements, it takes a 30–36 nm step size when Cy3 is positioned in front of Myo5c (Figure 9A), and a 25–30 nm step size when Cy3 is positioned behind the Myo5c-DNA complex (Figure 9B). In the “Front-Cy3 model”, where the Cy3 is positioned in front of the Myo5c-HMM complex, the stepping events are always contributed by the leading Myo5c-HMM molecule (pathway a → c → d) but not by the trailing one (pathway a → b → d). In the “Rear-Cy3 model” where the Cy3 is positioned behind Myo5c-HMM complex, stepping events are contributed by both molecules. The dwell time of the “Front-Cy3 model” takes twice as long as that of the “Rear-Cy3 model”. Moreover, we did not detect any large steps with 72-nm step-size, which would result from the rear Myo5c-HMM dimer flipping forward to the front of the complex. This indicates that the two Myo5c-HMM molecules on a DNA scaffold do not change their position during a processive run.

Lipowsky's group proposed a cargo transport model with two motor molecules^49^. In this model, the detachment rate and velocity depend on the internal strain between two motor molecules, and the stiffness of the linker between the two motors affects the run length and velocity. If the linker is stiff enough (e.g. strong coupling) compared with the internal strain caused by myosin's power stroke, the detachment rate for one of the two molecules will increase and become less processive. Hence, the velocity and run length of the complex will decrease. If the linker is sufficiently elastic compared to the internal force (e.g. weak coupling), the run length and velocity of the motor complex will remain unchanged. Our result showed that the velocity and run length of two Myo5c-HMM molecules on a DNA scaffold decreased with shorter distance between motors. This suggests a strong coupling model for two Myo5c-HMM molecules on a DNA scaffold. However, Myo5c-HMM-dDNA_36_ shows two types of stepping, in which the dwell times differ by a factor of two (Figure 7C and D). To explain the two stepping behavior, we propose that a rear Myo5c-HMM molecule in Myo5c-HMM-dDNA_36_ can step forward despite the stiffness of the DNA linker (persistence length: 50 nm). Thus, according to the Lipowesky's model, motor-motor coupling in Myo5c-HMM-dDNA is likely to be between strong and weak coupling.

It is possible that the motor-motor distance affects the binding between myosin and actin as a result of the geometric arrangement of actin helical repeat and the attachment distance on Myo5c-HMM-dDNA. Our results show that the shorter motor-motor distance decrease the run length, velocity, and the stepping manner of Myo5c-HMM-dDNA. Thus, this structural interference between two Myo5c-HMM molecules on a DNA scaffold can also contribute to disruption of the movement of two Myo5c-HMM molecules.

The kinetic mechanism of Myo5c is similar to the switch-1 mutation of myosin 5a^S217A^, in which the rate of phosphate dissociation is reduced approximately 10-fold to 20 s^−1^^50^, except that the phosphate dissociation in Myo5c is ~5 times slower. Similarly, myosin5a^S217A^ does not move processively at physiological ATP concentrations (>1 mM), though it displayed processive movement at lower ATP concentrations. However, the gating of ADP dissociation between the front and rear heads was maintained and remained very similar to Myo5c and myosin 5a^32^.

It is interesting to note that the maximum phosphate release rate from actoMyo5c-HMM(-ADP-Pi) is 2.1 times faster than from actoMyo5c-S1-ADP. The observed rate of phosphate dissociation increases with increasing actin concentration (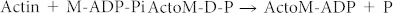) up to the maximum rate of phosphate dissociation (k'_4_) (Figure 2D). The two heads of Myo5c-HMM would decrease the rate that Myo5c-HMM (ADP-Pi)_2_ dissociates from actin and make the K_act_ of Myo5c-HMM smaller (Figure 2D). If phosphate dissociates from both heads of Myo5c-HMM (ADP-Pi)_2_, in which both heads have bound products, ADP and Pi at the same rate, then the observed rate that phosphate dissociates from actoMyo5c-HMM-(ADP-Pi)_2_ would be twice as fast as that of Myo5c-S1-ADP-Pi. The result suggests that phosphate dissociation from each head of actoMyo5c-HMM-(ADP-Pi)_2_ is not an ordered mechanism and is independent of each head.

Myosin 5a in which a single α-helix (SAH) domain replaces four of the six IQ domains moves processively along actin at physiological ATP concentrations with similar stride and run lengths to a wild type myosin 5a with 6IQs in *in vitro* single molecule assays^51^. The result demonstrates that the key property necessary to produce a processive myosin motor is that after the trailing head is dissociated by ATP, it must reattach to actin to form a strongly bound AM-ADP at a rate that is considerably more rapid than the rate that ADP dissociation and ATP binding occur on the other head. Although the slow rate of phosphate dissociation from actomyosin 5c-ADP-Pi relative to the rate of ADP dissociation prevents it from functioning as a processive motor, this shortfall can be circumvented by utilizing multiple myosin molecules to reduce the odds of all of the heads being dissociated at once.

In summary, we successfully engineered a complex consisting of two coupled Myo5c-HMM dimers and demonstrated the processive movement of this low-duty-ratio myosin. Our study will provide molecular insight into the biological functions of Myo5c in cells.

## Methods

### Constructs, Proteins and Reagents

The plasmid containing the cDNA sequence of full length human Myo5c was provided by Dr. Cheney, University of North Carolina at Chapel Hill. The cDNAs encoding Myo5c subfragment 1 (Myo5c-S1, 1–785 amino acids) and heavy meromyosin fragment (Myo5c-HMM, 1–1109 amino acids) were amplified by PCR and sub-cloned into pFastBac1 vector (Invitrogen). The Flag epitopes (DYKDDDDK) were engineered into the vectors before the stop codon. To generate Myo5c-HMM with a SNAP-tag, which is used for linking Myo5c-HMM fragments to the amine-modified DNA scaffolds, the cDNA encoding SNAP (snap26b, New England Biolabs) was inserted immediately after Myo5c-HMM followed by either a FLAG tag or six repeated histidines (6x-His) before the stop codon. All the constructs were verified by DNA sequencing. Myo5c-S1 and -HMM proteins were expressed using the baculovirus Sf9 system and purified by anti-FLAG affinity chromatography as described previously^52^.

To generate heterodimer Myo5c-HMM with single SNAP-tag, which has a SNAP-tag with a FLAG-tag on one heavy chain and 6x-His tag on the other. Myo5c-HMM-SNAP-FLAG and Myo5c-HMM-6x-His were co-expressed with calmodulin in Sf9 cells. This would be expected to produce a mixture of two homodimers, Myo5c-HMM-His/Myo5c-HMM-6His and Myo5c-HMM-SNAP-FLAG/Myo5c-HMM-SNAP-FLAG, and one heterodimer Myo5c-HMM-6His/Myo5c-HMM-SNAP-FLAG. The heterodimer was purified from the mixture of expressed proteins as follows: First, Ni-NTA agarose (Invitrogen) was mixed with soluble Myo5c-HMM solution and eluted with 20 mM Na-Pi (pH 7.5), 1 mM MgSO_4_, 250 mM NaCl, 1 mM DTT, and 250 mM Imidazole. The eluted solution was concentrated with an ionic exchange column (Q Sepharose) and dialyzed by changing the buffer (0.1 mM EGTA, 20 mM MOPS pH 7.3, 1 mM MgCl_2_. 40 mM KCl, 0.1 mM PMSF, and 5 mM DTT) three times every three hours. The dialyzed Myo5c-HMM solution containing Myo5c-HMM-His/Myo5c-HMM-6His and heterodimer Myo5c-HMM-6His/Myo5c-HMM-SNAP-FLAG was then applied to an anti-FLAG-affinity chromatography column, followed by washing with the dialysis buffer equivalent to five times the volume of the column. The heterodimer Myo5c-HMM-6x-His/Myo5c-HMM-SNAP-FLAG was eluted by FLAG peptide with an elution buffer and concentrated using a Q-sepharose column.

Rabbit skeletal muscle was purchased from Pel-Freez Biological, and actin was prepared as described previously^53^. MDCC-PBP, *N*-[2-(1-maleimidyl) ethyl]-7-diethylaminocoumarin-3-carboxamide labeled phosphate-binding protein, deac-amino ADP and ATP were prepared according to published procedures^54^.

### Actin-activated ATPase activity assay

The steady state actin-activated ATPase was measured using the NADH-coupled assay in the buffer containing 10 mM MOPS, pH 7.4, 2 mM MgCl_2_, 40 mM KCl, 0.1 mM EGTA, 2 mM ATP, 40 units/ml lactate dehydrogenase, 200 units/ml pyruvate kinase, 1 mM phosphoenolpyruvate, and 200 mM NADH^45^. The change in NADH absorption at 340 nm (molar extinction coefficient (ε): 6220 M^−1^cm^−1^) was monitored in 300 μl cells by a spectrophotometer (Model: Cary50, Agilent). The ATPase activity of blanks containing actin filament without myosin, was subtracted from each experiment to determine the steady state rate.

### Preparation of DNA scaffold and myosin-DNA complex

The nucleotide sequence used in this study was designed by computer simulation as described^55^. The DNA sequences for 6.8-nm, 18-nm, and 36-nm separation distance were 5′-T[Amino-C6dT]CTTGGCCGAACTGAAGTGA[Amino-C6dT]CCAGCTTATAGATATGGGCACGTAAACAAGCAT-3′, 5′-[Amino]TCTTGGCCGAACTGAAGTGATCCAGCTTATAGAT ATGGGCACGTAAACAAGCAT[Amino]3′, and 5′-[Amino] TTCTTGGCCGAACTGAAGT GATCCAGCTTATAGATATGGGCACGTAAACAAGCATCCGTTGGTCTAGGAGTAGTTACAATTCCCCGGTTCCGCTCATTTCGATT-[Amino]3′, respectively ([Amino-C6dT] denotes internal amino modification). The double-stranded DNA scaffold was synthesized with one strand labeled with fluorescent dye Cy3 at the 5′ end, and the other strand with amine at both ends for 18-nm and 36-nm and at internal amino modification for 6.8-nm. The amine-modified strands were incubated with the SNAP-tag substrate benzylguanine (BG-GLA-NHS, New England BioLabs) at 37°C overnight. The benzylguanine-modified DNA was separated from the unmodified DNA using Micro Biospin columns (Bio-Rad).

To conjugate two double-headed Myo5c on a DNA scaffold, the heterodimer Myo5c-HMM-SNAP was incubated with the modified DNA scaffold at a molar ratio of 1:5 (DNA: Myo5c-HMM-SNAP) at 27°C for 1 hour, and the free Myo5c molecules were removed by gel-filtration column (S-400 sepharose, FPLC AKTA, GE Healthcare). The eluted Myo5c-HMM-DNA scaffold solution was then concentrated by spin-column (30 k pore size, TransGloba Scientific Inc.).

### Total internal reflection fluorescence (TIRF) motility assay

Single molecule motility assays were performed as previously described^40^. Cy3-labeled DNA scaffold conjugated with two Myo5c-HMM dimers or Alexa Fluor 568-labeled Myo5c-HMM, and Alexa Fluor 488-phalloidin labeled actin filaments were imaged under a TIRF microscope. For Alexa Fluor 488-actin filaments, we used a narrow emission filter (ET 510/25 nm bandpath filter, Chroma Technology corp.). The particle localization was achieved by Fluorescence Imaging with One Nanometer Accuracy (FIONA)^41^. The tracking of fluorescent spots was analyzed using custom IDL software^52^. The velocity and run-length of the processive runs were calculated by Origin lab.

### ADP dissociation kinetics assay of myo5c-HMM and -S1

Transient kinetic measurements were performed with A SFM-300 stopped-flow (Biologic USA) equipped with a μSf-8, 4 μl micro cuvette using software provided by the manufacturer. The dead-times were 0.25 msec for a total flow-rate of 13.3 ml/s and 1 msec for a total flow-rate of 4 ml/s. Experimental conditions were 20 mM MOPS pH 7.3, 5 mM MgCl_2_, 40 mM KCl, 0.1 mM EGTA, 23°C. All data were obtained from averages of three or four traces. Three different purifications of Myo5c protein were examined. The data were presented as average and standard deviation (S.D.) for all solution kinetics studies.

Double mixing reactions were used to measure the ADP dissociation from Myo5c-HMM or S1. Myosin solutions (0.5 μM, 100 μl) in syringe 1 and ATP (2 μM, 100 μl) in syringe 2 were mixed and incubated for 20 sec in a delay-line allowing ATP to bind to and be hydrolyzed by Myo5c. Next, the solution was mixed in one-to-one ratio with 40 μM phalloidin F-actin and excess deac-aminoADP (1 mM) in syringe 3 to prevent re-binding of dissociated ADP. Actin filaments were stabilized by equimolar phalloidin. Stock solution of ADP (2 mM) and phalloidin-F-actin (100 μM) were treated for 1 hour at 20°C with 1 mM glucose and 0.01 unit/ml hexokinase to remove traces of ATP in experiments. To observe deac-aminoADP dissociation, deac-aminoATP in syringe 2 was mixed with Myo5c proteins in the first mix, and mixed with phalloidin F-actin and excess ADP (1 mM) in syringe 3 after 20 sec. Deac-aminoADP and deac-aminoATP were excited at 442 nm wavelength. The emitted light was filtered with a 455 nm long pass filter and was acquired by positioning the detector at 90° to the excitation path.

The fitting of the experimental data to the time course was analyzed using software provided by Bio-logic USA (Bio-kine) and Origin Pro ver. 8.5 (Origin lab). Each data point was averaged over 4–6 traces and was fitted to a single- or double-exponential equation as follows: 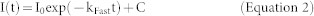

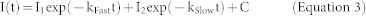


I(t) is fluorescence signal over time t. k_Fast_ and k_Slow_ are the apparent rates. I_0_, I_1_, and I_2_ are the corresponding amplitude coefficients of the fast and slow exponential parts.

### Phosphate release kinetics assay of Myo5c-HMM

Phosphate dissociation from actomyosinADP-Pi complex was measured using fluorescently labeled phosphate binding protein MDCC-PBP as described^54^,^56^. To remove ATP, actin (100 μM) and ADP (1 mM) solutions were treated with 1 mM glucose and 0.01 unit/ml hexokinase for 1 h at 20°C. All working solutions contained 6 μM MDCC-PBP and the phosphate-mop, containing 0.1 mM 7-methylguanosine and 0.01 units/ml purine-nucleoside phosphorylase.

Myo5c-HMM or -S1 (1.2 μM, 71 μl) in syringe 1 was first mixed with ATP (2 μM, 71 μl) in syringe 2 and incubated for 5 sec in a delay-line. Then, the Myo5c-HMM-ATP or Myo5c-S1-ADP-Pi complex (25 μl) was mixed with varying concentrations of phalloidin-stabilized F-actin (25 μl) in syringe 3. The time course of phosphate release was fitted with a single exponential function.

## Author Contributions

L.K.G., K.F., J.B. and T.S. designed research, performed experiments, analyzed data, and wrote the manuscript. H.K. and H.D.W. designed the research and wrote the manuscripts. M.K.U. measured actin-activated ATPase activity.

## Supplementary Material

Supplementary Information Supplemental information

Supplementary Information Movie 1

Supplementary Information Movie 2

Supplementary Information Movie 3

Supplementary Information Movie 4

## Figures and Tables

**Table 1:** Actin Activated ATPase

Myosin	V_max_ (S^−1^)	K_ATPase_ (μM)
Myo5c-S1	2.76 ± 0.65	26 ± 3.7
Myo5c-HMM	2.31 ± 0.55	5.1 ± 1.8
Myo5c-HMM-SNAP	2.37 ± 0.48	4.8 ± 2.1

Data: mean ± S.D.

**Table 2:** ADP/Deac-aminoADP dissociation rate from actomyosin Vc

Myosin	Monitoring	Chasing with	Fast rate (s^−1^)	Slow rate (s^−1^)
Myo5c-S1	ADP dissociation	Deac-aminoADP	14.3 ± 0.3	N/A
Myo5c-S1	Deac-aminoADP dissociation	ADP	1.25 ± 0.21	N/A
Myo5c-HMM	ADP dissociation	Deac-aminoADP	5.5 ± 0.2	0.028 ± 0.009
Myo5c-HMM	Deac-aminoADP dissociation	ADP	0.31 ± 0.02	0.0086 ± 0.0021
Myo5c-HMM	ADP dissociation	Deac-aminoATP	3.9 ± 0.4	N/A
Myo5c-HMM	Deac-aminoADP dissociation	ATP	0.29 ± 0.09	N/A
Myo5c-HMM-SNAP	ADP dissociation	Deac-aminoADP	5.8 ± 0.2	0.021 ± 0.01
Myo5c-HMM-SNAP	Deac-aminoADP dissociation	ADP	0.28 ± 0.05	0.0106 ± 0.0021
Myo5c-HMM-SNAP	ADP dissociation	Deac-aminoATP	4.2 ± 0.3	N/A
Myo5c-HMM-SNAP	Deac-aminoADP dissociation	ATP	0.21 ± 0.09	N/A
Myo5c-S1*^1^	mant-ADP dissociation	ATP	16 ± 0.6*^1^	N/A
Myo5c-S1*^2^	dmant-ADP dissociation	ADP	17.7 ± 0.6*^2^	N/A

*1: Ref 55, Takagi Y., et al., 2008, *J. Biol. Chem.*, 283, 13, 8527.

*2: Ref 56, Watanabe T., et al., 2008, *J. Biol. Chem.*, 283, 16, 10581.

Date: mean ± S.D., N/A; not available.
