# Supplementary material for: Coupling of Two Non-processive Myosin 5c Dimers Enables Processive Stepping along Actin Filaments
Source: Sci Rep. 2014 May 9;4:4907. doi: 10.1038/srep04907 (PMC4014986; doi:10.1038/srep04907)
Supplement: Supplementary Information — Supplemental information [file srep04907-s1.pdf]

Title: Coupling of Two Non-processive Myosin 5c Dimers Enables Processive Stepping along Actin Filaments

Authors:

Laura K. Gunther[1], Ken'ya Furuta[3], Jianjun Bao[1], Monica K. Urbanowski[1], Hiroaki Kojima[3], Howard D. White[4] and Takeshi Sakamoto[1,2,*]

Materials and Methods

Actin gliding assay

The actin gliding assay was performed as described in a previous study[1]. An ATP regeneration system containing 100 units/ml pyruvate kinase and 0.1 mM phosphoenolpyruvate was added to the motility buffer (40 mM KCl, 20 mM MOPS pH 7.3, 5 mM $MgCl_2$, 0.1 mM EGTA, 5 mM dithiothreitol (DTT)). The velocity of rhodamine-phalloidin labeled F-actin was measured by MetaMorph (Molecular Device) and Origin 8.5 (OriginLab).

Movie-1

Attachment and dissociation of single Myo5c-HMM molecule on actin filaments. All movies were captured by three frames/second and are 4 times faster than real speed.

Movie-2

The movement of Myo5c-HMM-DNA_{36} on actin filaments.

Movie-3

The movement of Myo5c-HMM-DNA_{18} on actin filaments.

Figures

Supplemental Figure S1.

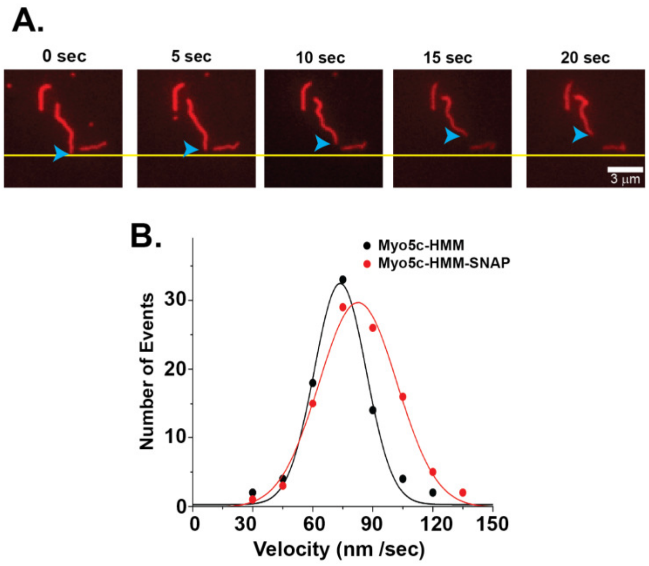

Supplemental Figure S1. (A) Sequential images of actin gliding over Myo5c-HMM. Yellow lines represent the starting point of the movement of the actin filament. Actin filaments were labeled by Rhodamine-Phalloidin (red). Blue arrow heads represent the position of the end of the actin filament. Scale bar: 3 µm. (B) Histogram of the velocity of actin filaments for Myo5c-HMM with (black circles) and without (red circles) SNAP tag. The data were fit with a single Gaussian function. The average velocities (means ± S.D.) of Myo5c-HMM and Myo5c-HMM-SNAP were 73.7 ± 15 (n = 77) and 82.6 ± 26 (n = 97) nm/s, respectively.

Supplemental Figure S2.

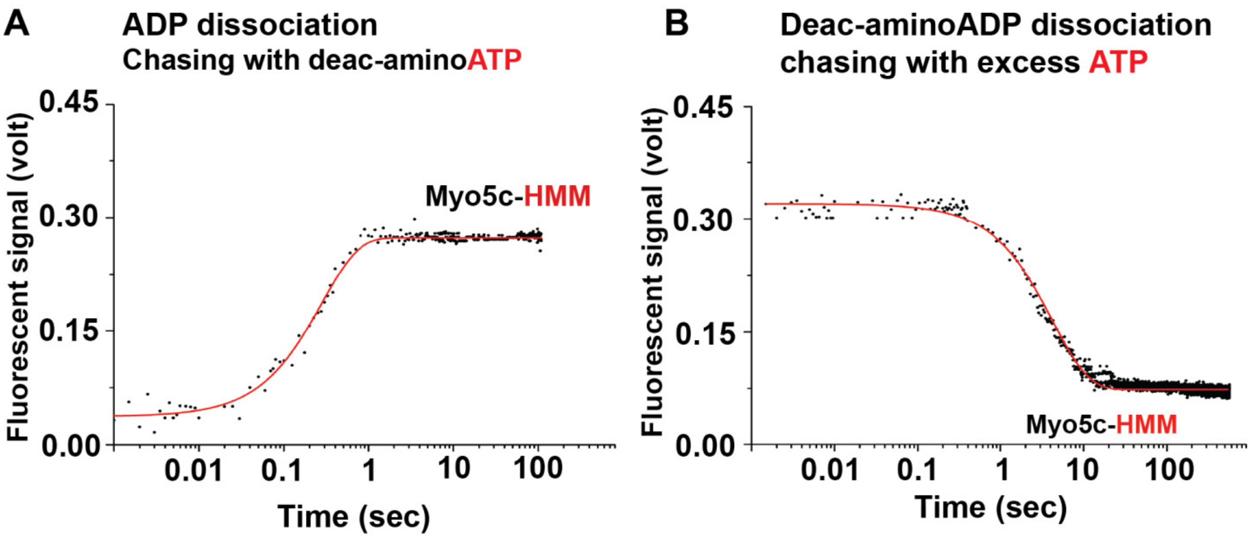

Supplemental Figure S2. ADP dissociation and deac-aminoADP dissociation from actomyosin 5c-HMM by excess deac-aminoATP (800 µM) or ATP (1 mM). (A) ADP dissociation from actomyosin 5c-HMM with excess deac-aminoADP (800 µM). The data are the average of three traces and were fit with single exponential function (red line) with a rate of 13.5 ± 1.1 s^{-1} and an amplitude of 0.26. (B) Deac-aminoADP dissociation from actomyosin 5c-HMM with excess ATP (1 mM). The data are the averaged of three traces and were fit with a single exponential funtion (red line) with a rate of 0.29 ± 0.09 s^{-1} and an amplitude 0.27.

Supplemental FigureS3

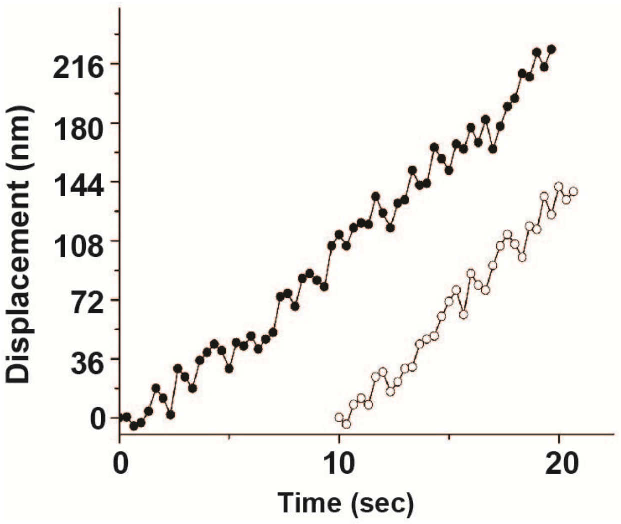

Supplemental Figure S3. Stepping traces of Cy3-Myo5c-HMM-dDNA$_{6.8}$.

1 Sakamoto, T. *et al.* Neck length and processivity of myosin V. *J Biol Chem* **278**, 29201-29207, doi:10.1074/jbc.M303662200

M303662200 [pii] (2003).
